# The effect of texture and grain size on improving the mechanical properties of Mg-Al-Zn alloys by friction stir processing

**DOI:** 10.1038/s41598-018-22344-3

**Published:** 2018-03-08

**Authors:** Jinhua Peng, Zhen Zhang, Zhao Liu, Yaozu Li, Peng Guo, Wei Zhou, Yucheng Wu

**Affiliations:** 1 School of Materials Science and Engineering, Hefei University of Technology, Hefei, 230009 China; 2 National–Local Joint Research Center of Non-ferrous Metal Materials and Processing Technology, Hefei, 230009 China

## Abstract

Friction stir processing (FSP) was used to achieve grain refinement on Mg-Al-Zn alloys, which also brought in significant texture modification. The different micro-texture characteristics were found to cause irregular micro-hardness distribution in FSPed region. The effects of texture and grain size were investigated by comparative analyses with strongly textured rolling sheet. Grain refinement improved both strength and elongation in condition of a basal texture while such led to an increment in yield stress and a drop in elongation and ultimate stress when the basal texture was modified by FSP.

## Introduction

Magnesium (Mg) alloys gained increasing interest for aerospace and automotive industries in the past few decades due to their prominent performance in weight saving. However, due to the limited number of available slip systems in the hexagonal closed-packed (HCP) structure, Mg alloys show poor formability especially at lower temperature^1^. This was widely believed to be one of the most important factors to restrict their wider application in industry. It has been frequently reported that grain refinement could effectively improve the mechanical properties of Mg alloys, both in strength and plasticity^2,3^. Moreover, the grain size strengthening efficiency was reported to be higher than Al or other alloys^4^. For the above reasons, massive effort has been made to obtain ultrafine grain structure for Mg alloy^5–8^.

Among all grain refining techniques, severe plastic deformation (SPD) method was considered an industrial reliable, controllable and relatively inexpensive route compared to other methods, such as rapid solidification^9–11^, etc. However, other effects from SPD, such as texture modification would also have potential influence on mechanical properties of metallic materials, especially for magnesium alloys, which shows obvious anisotropy^12–14^. The influence of SPD on the mechanical properties of Mg alloys has been frequently reported, including equal-channel angular pressing (ECAP)^15,16^, friction-stir processing (FSP)^17,18^, high-pressure torsion (HPT)^19,20^, accumulative rolling (AR)^21,22^, etc. However, the net effect from grain refinement and texture was still not well investigated due to the complex texture condition that was introduced by different processing routes.

Here we chose friction-stir processing (FSP) to achieve grain refinement, because it was an effective technique to conduct on sheets products, which were most commonly used types for magnesium alloys. Previous study well revealed the influence of FSP parameters on the post-processed microstructure and texture^23–25^; a {0002} B-fiber basal texture was always obtained after FSP^26^. R.S. Mishra^27^ and J.C. Huang^28^ claimed that a lower friction stress and H–P slope was obtained in condition of FSP induced texture modification. Extensive studies^18,29,30^ show that the mechanical properties were improved, especially for ambient ductility along processing direction (PD), due to the combined effect from texture modification and grain refinement. However the individual effect from texture and grain size was not fully understood. In the present work, strongly textured rolled sheets and FSPed material with different grain sizes, as well as single crystals, were used for comparative analyses to reveal the separate effect from texture and grains size on the mechanical properties for the experimental material. Moreover, the micro-texture related micro-hardness distribution in FSPed region was also thoroughly studied.

## Experimental Methods

The material used in current study was hot-rolled commercial AZ31 Mg alloy plate, 3 mm and 5 mm in thickness. The nominal chemical composition (wt.%) was 3.0% Mg, 1.0% Zn and the balance Mg. A stirring tool with a 15 mm-diameter shoulder and 3.5 mm-conical pin was used for FSP, which was conducted with different parameters to prepare different grain sizes. For comparison, 3 mm hot-rolled plates were annealed at different temperatures varying from 300 °C to 500 °C to prepare different grain sizes.

Both metallographic observation and EBSD measurements were conducted on the ND-TD plane (Normal direction- Transverse direction) of the FSPed sample. Macrotexture was measured on the PD-TD plane (Processing direction-Transverse direction) for FSPed samples and on the RD-TD plane (Rolling direction-Transverse direction) for rolling sheets, with Panalytical X-ray diffractometer in Schulz reflection geometry.

Vickers micro-hardness was measured with 300 g load for 10 s. Dog-bone shaped tensile specimens (16 mm gage length, 4 mm gage width and 1 mm gage thickness) were cut along processing direction (PD) from the top surface of FSP sample by electrical discharge machining. Tensile tests were carried out with a crosshead speed of 1 mm/min at room temperature.

## Results and Discussion

### Microstructure observation

**Figure 1:**
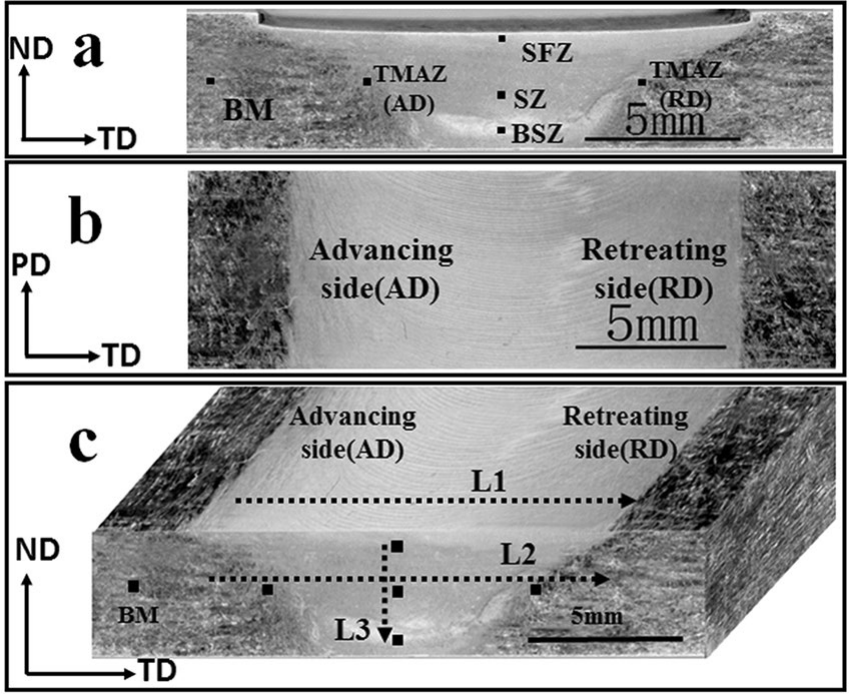
Low-magnification overviews of the (**a**) ND-TD plane, (**b**) PD-TD plane and (**c**) the three-dimensional picture of FSPed sample.

**Figure 2:**
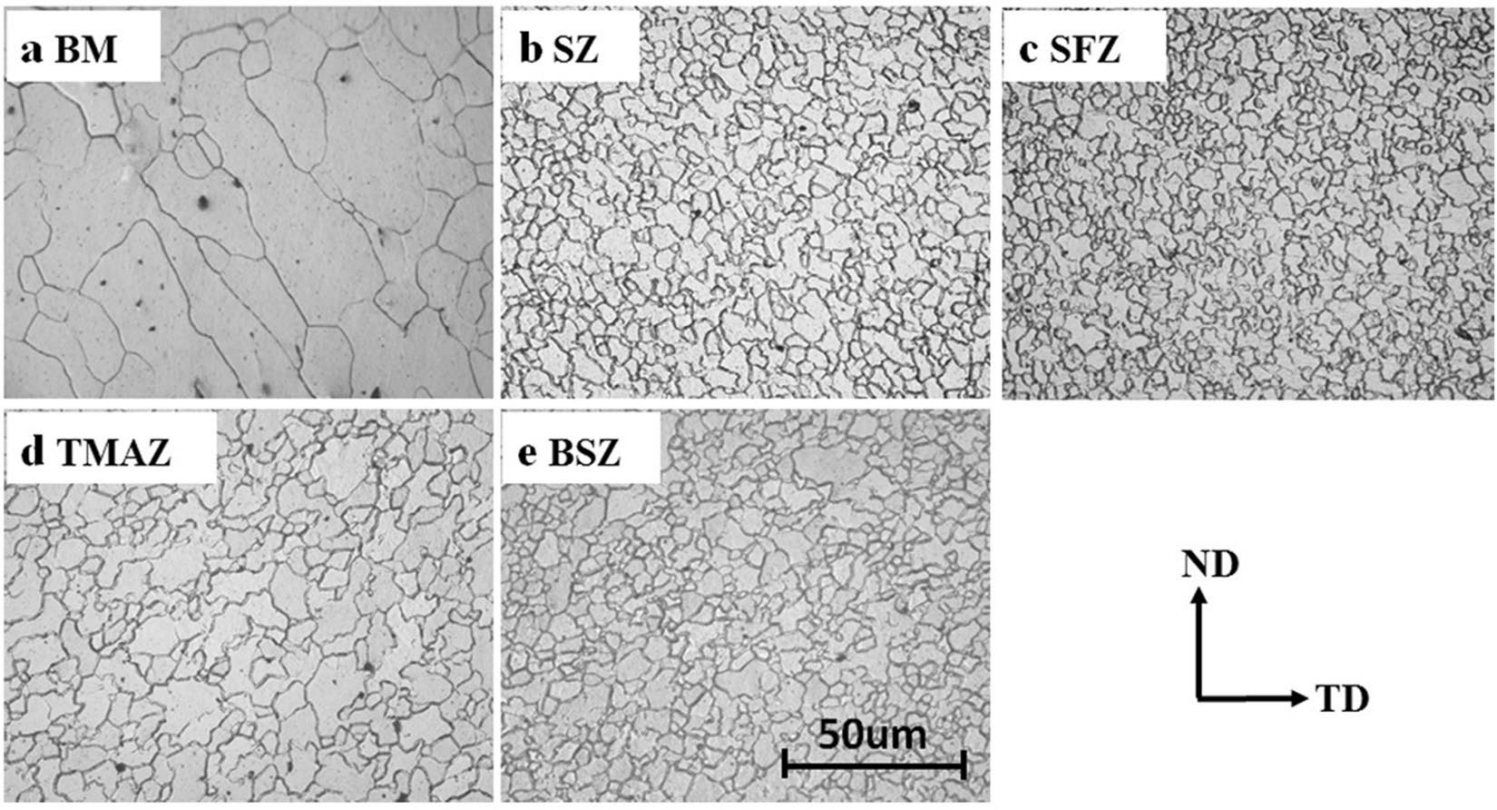
The optical micrographs of (**a**) base material (BM), (**b**) stir zone (SZ), (**c**) shoulder-affect zone(SFZ), (**d**) thermo-mechanically affected zone (TMAZ) and (**e**) bottom of stir zone (BSZ) of FSPed sample.

**Figure 3:**
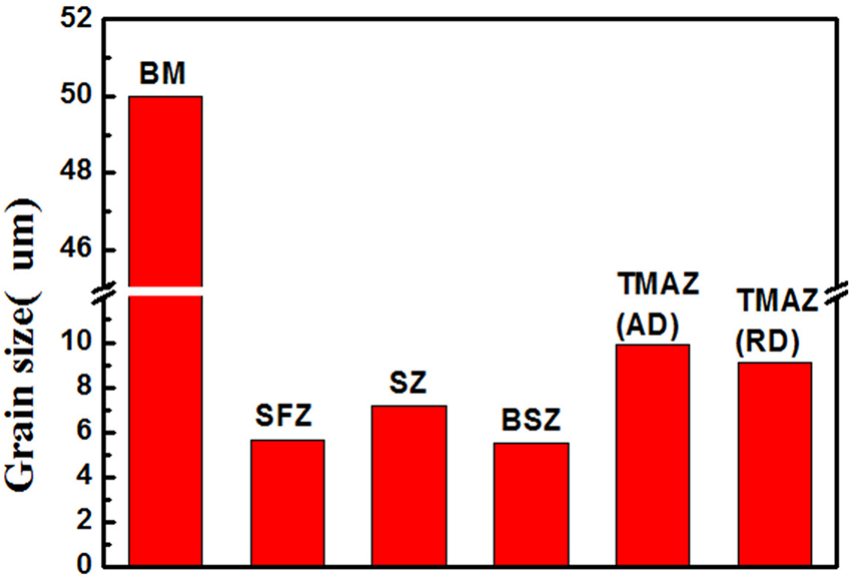
The grain size distribution of various zones for FSPed sample.

Figure 1 shows the macrostructure of FSPed specimen (1200 rpm–60 mm/min). A cone-shaped processed region could be easily distinguished, which could be mainly divided into three different regions - stir zone (SZ), thermo-mechanically affected zone (TMAZ) and base material (BM). The typical microstructures were shown in Fig. 2a–e. It could be clearly seen that the BM showed coarse grains with an average size of about 50 um; and much smaller grains were produced in SZ region, which implied the occurrence of complete dynamic recrystallization (DRX) during FSP. However, the grain size shows some variation from the top surface (shoulder-affect zone, SFZ) to the bottom of stir zone (BSZ), where the flow of the material was more influenced by the pressure of shoulder and the rotating state of stir pin respectively. On the other hand, TMAZ showed more complicated microstructure characterized by elongated grains with serrated boundaries. The average grain sizes in different regions were shown in Fig. 3. In spite of some variation in different location, it was clearly shown that significant grain refinement was achieved by FSP.

### Micro-hardness distribution in FSPed region

**Figure 4:**
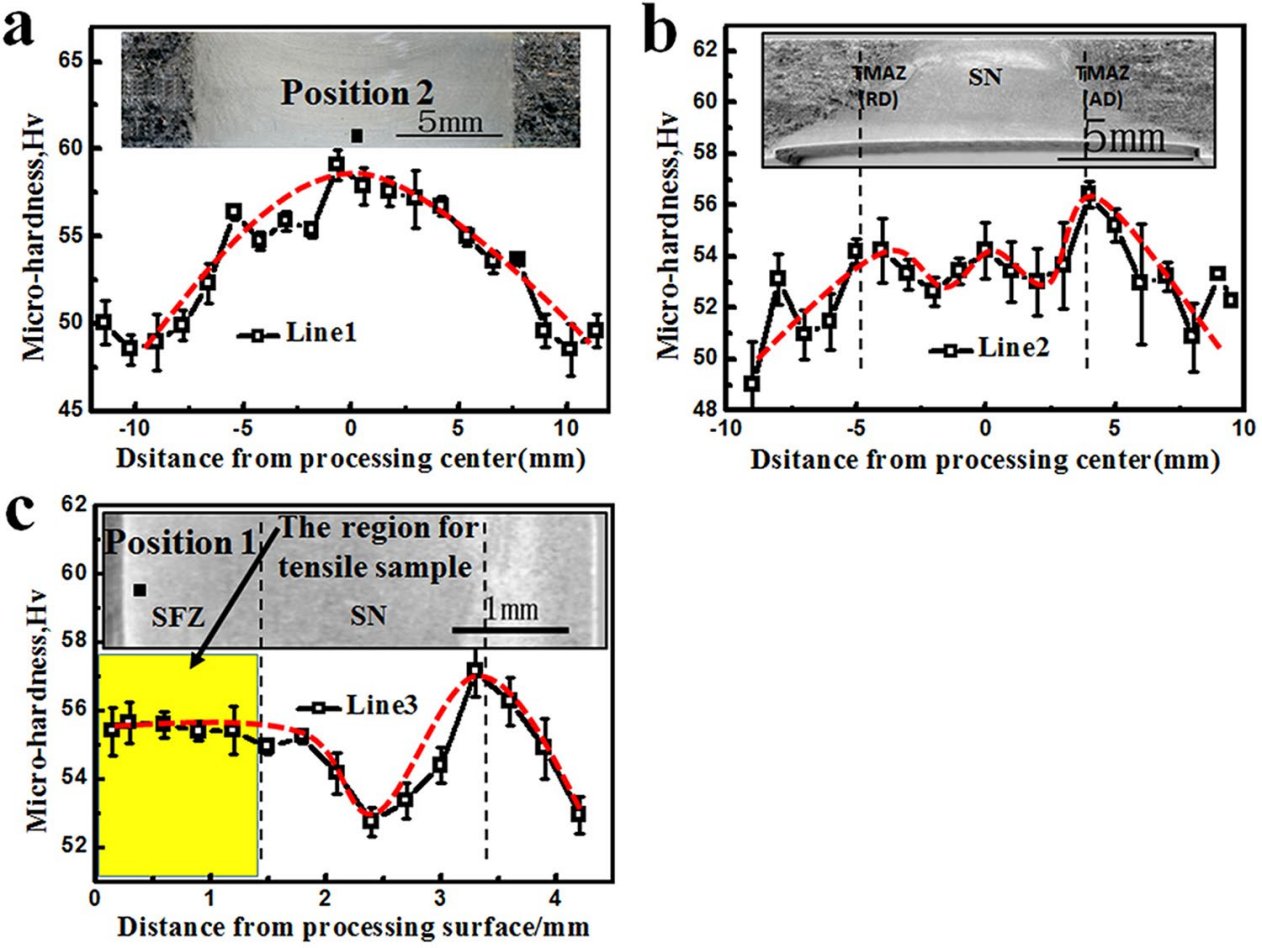
The micro-hardness distribution of FSPed sample along (**a**) L1, (**b**) L2 and (**c**) L3.

In order to investigate the influence of FSP on mechanical properties of the experimental material, firstly the distribution of micro-hardness along L1, L2 and L3 (marked in Fig. 1c) were measured, as shown in Fig. 4a–c. All these results invariably showed that the hardness was higher in FSPed region than in BM, which might be attributed to the grain refinement effect caused by DRX (also well known as Hall-Petch effect). However, the micro-hardness was not quite uniformly distributed. Although the measured value scattered slightly to an extent, general trend could still be seen. Firstly, the micro-hardness gradually increased when approaching the centerline on the PD-TD and ND-TD plane. Secondly, although the grain size was of similar level along L3 line on the ND-TD plane, the hardness was not all uniform and an evident hardness valley was found lying in the middle of SZ region. Thirdly, the measured value seemed to present some deviation on different tested planes, even for the same areas. Take the position in the middle of SFZ region for example. The hardness was measured to be 55HV and 59HV respectively for Position No. 1 and 2 (as shown in Fig. 4a,c) on ND-TD and PD-TD planes, which in fact represented the same region in the middle of SFZ.

**Figure 5:**
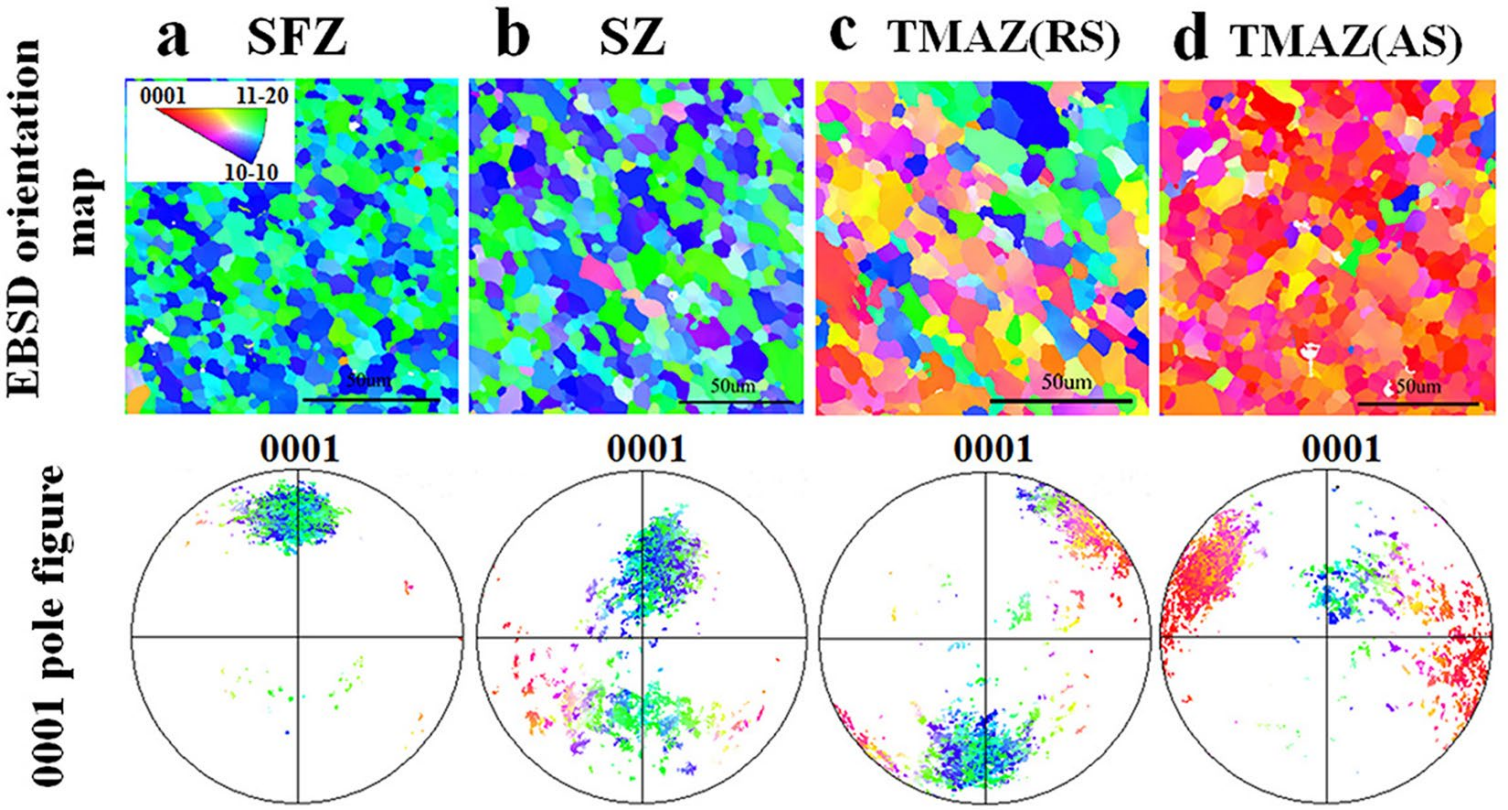
EBSD orientation maps and 0001 pole figures illustrating microstructure and texture in (**a**) SFZ, (**b**) SZ, (**c**) TMAZ (RS) and (**d**) TMAZ (AS) of FSPed sample.

**Figure 6:**
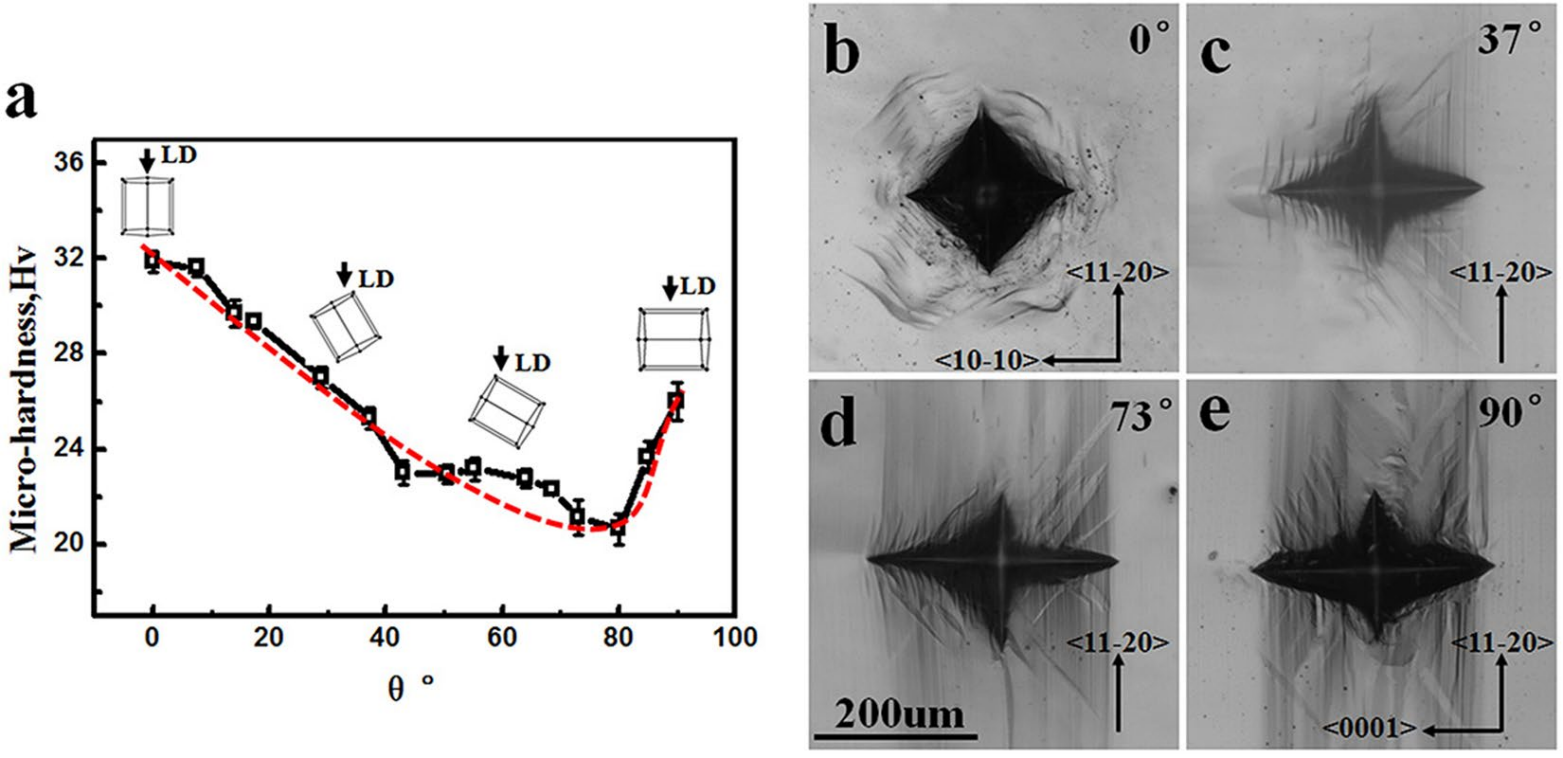
(**a**) The micro-hardness evolution of single crystal with the increase of angle θ between <0001> and loading direction (LD) and the morphology of indentations at (**b**) 0°, (**c**) 37°, (**d**) 73° and (**e**) 90°.

Since the grain size did not show much variation within the processed region, the Hall-Petch effect did not seem to play an important role in the present case. Thus the observed irregular hardness distribution in FSP region might be attributed to other factors, more probably from micro-texture effect. In order to reveal such possible effect, EBSD measurements were conducted on the cross-section of FSP region and the results are shown in Fig. 5a–d. As a guideline, the micro-hardness was also tested from different orientations on a single crystal; the corresponding indentation was examined by optical microscopy (Fig. 6). It could be clearly seen that the micro-hardness showed the highest value when loading direction was along c-axis thus suppressing basal slip. However, some thick twins were also observed near the indentation, which might be formed due to inevitable lateral squeeze from the indenter. With the increase of inclined angle between loading direction and c-axis, the hardness value gradually decreased due to easy activation of basal slip. Plenty of basal slip traces and some twins could be found near indentation when the loading orientation was 37° and 73° (Fig. 6b,c). It was worth noting that the twins show different morphology from that of 0° orientation. To higher angle, when the tendency of basal slip got weakened and $\{10\bar{1}2\}$ twinning started to be involved, the micro-hardness underwent a slight increment.

EBSD results showed that the micro-texture was not uniform within FSP zone probably due to the different flow characteristics of material during FSP. The {0002} poles in SFZ region inclined around 20° from ND to PD; and this inclined angle gradually increased from SFZ region (surface) downwards to SZ region (bottom). This might explain the gradual decrease in micro-hardness along L3 line from the working surface to the bottom on the ND-TD plane, based on the hardness results from single crystal as described above. Furthermore, the {0002} poles near TMAZ (RS and AS) region inclined around 45° from ND to TD, which indicated the easiest orientation for dislocation slip and somewhat lower micro-hardness, so a gradual increase of hardness was revealed when measured from the edge to the processing centerline on the PD-TD plane. It need point out here that the orientation induced hardness variation was actually not so high (<10 HV) in the present case. This was consistent with Xin’s^31^ previous work, in which he concluded that the hardness was insensitive to texture variation; however a slight higher HV value was still measured for hard orientation. In our present work, by carefully controlling experimental errors we did also observe a general climbing trend in hardness when getting close to basal orientation. This could get convincing evidence from single crystals, where the maximum deviation reached 12 HV for different orientations.

### Mechanical properties from uniaxial tensile tests

**Figure 7:**
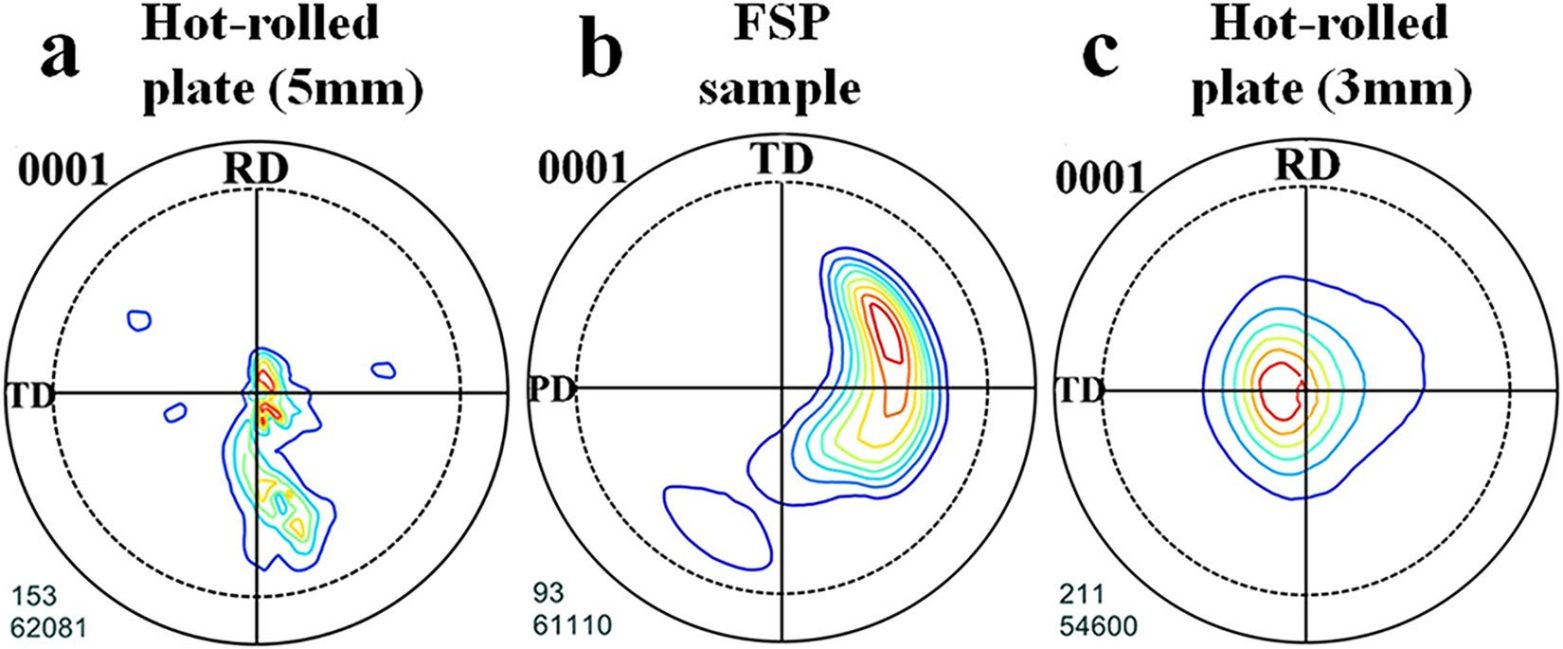
The X-ray pole figures show the macro-texture of (**a**) 5 mm hot-rolled plate, (**b**) FSPed sample and (**c**) 3 mm hot-rolled plate.

**Figure 8:**
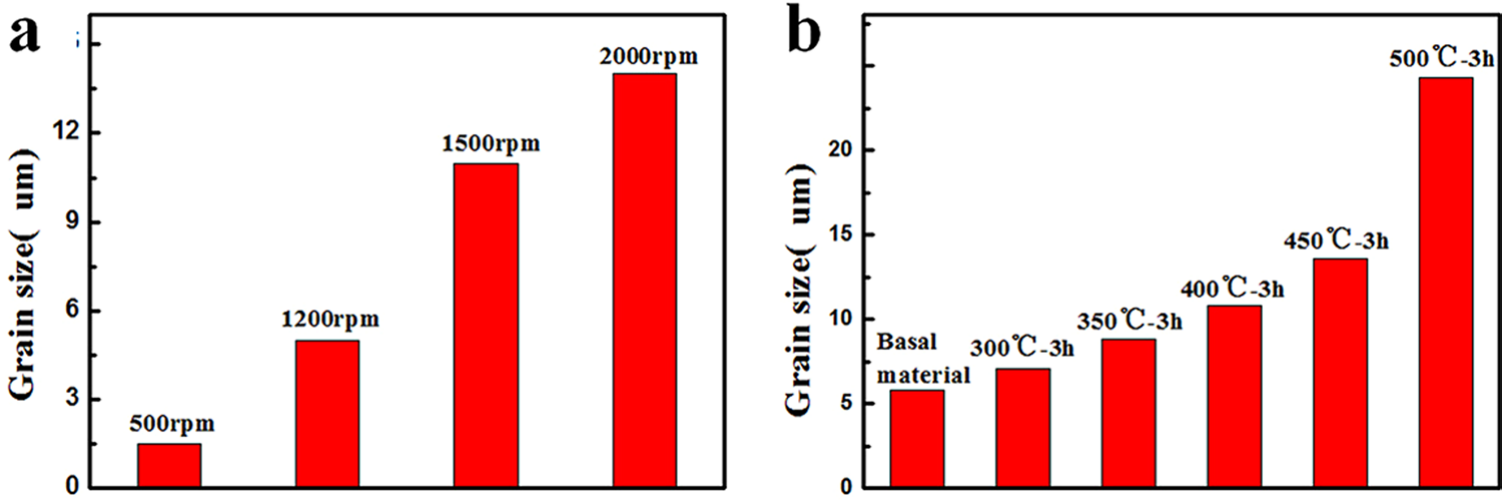
The grain size distribution of (**a**) FSPed samples with different rotational speed and (**b**) 3mm hot-rolled plates with different annealing temperature.

In the present work, the macro-texture was also measured by X-ray on the sheet plane for both the starting and FSPed samples as shown in Fig. 7a,b. It could be clearly seen that the starting material showed a typical basal texture; and after FSP the (0002) poles inclined 30°–50° from ND to PD-TD plane, which was almost consistent with the EBSD results for SFZ region. As stated above, although FSP could strengthen the material by Hall-Petch mechanism, the micro-texture was also an important factor affecting the micro-hardness of the different regions. To fully reveal the effect of FSP on mechanical properties, uniaxial tensile test were conducted on specimens cut from the SFZ area, where the grain size and micro-hardness was somewhat homogeneous (the region highlighted in yellow in Fig. 4c). It should be noted the grain size of FSPed samples increased from 1 um to13 um following the increase of rotating speed, the grain size distribution was shown in Fig. 8a.

**Figure 9:**
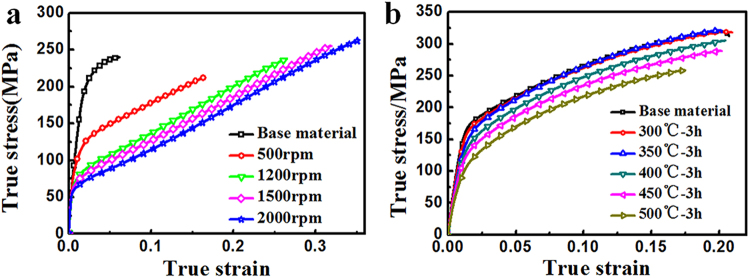
The true stress vs. true strain curves of (**a**) FSPed samples with different rotational speed and (**b**) hot-rolled plates with different annealing temperature.

The true stress- true strain curves were put together for comparison, as shown in Fig. 9a. It could be clearly seen that the most impressive improvement in mechanical properties that was obtained by FSP was the tensile elongation, which increased from 6% for BM to 16.6–35% after FSP. It needs to be stressed that the ultimate tensile strength also got a slight increase from 240 Mpa to 263 Mpa for the sample processed at 2000 rpm–60 mm/min; the only dark side was the evident loss in yield stress from 116 Mpa to 57 Mpa after FSP. Besides, the experimental material also shows different strain hardening characteristic after FSP.

**Figure 10:**
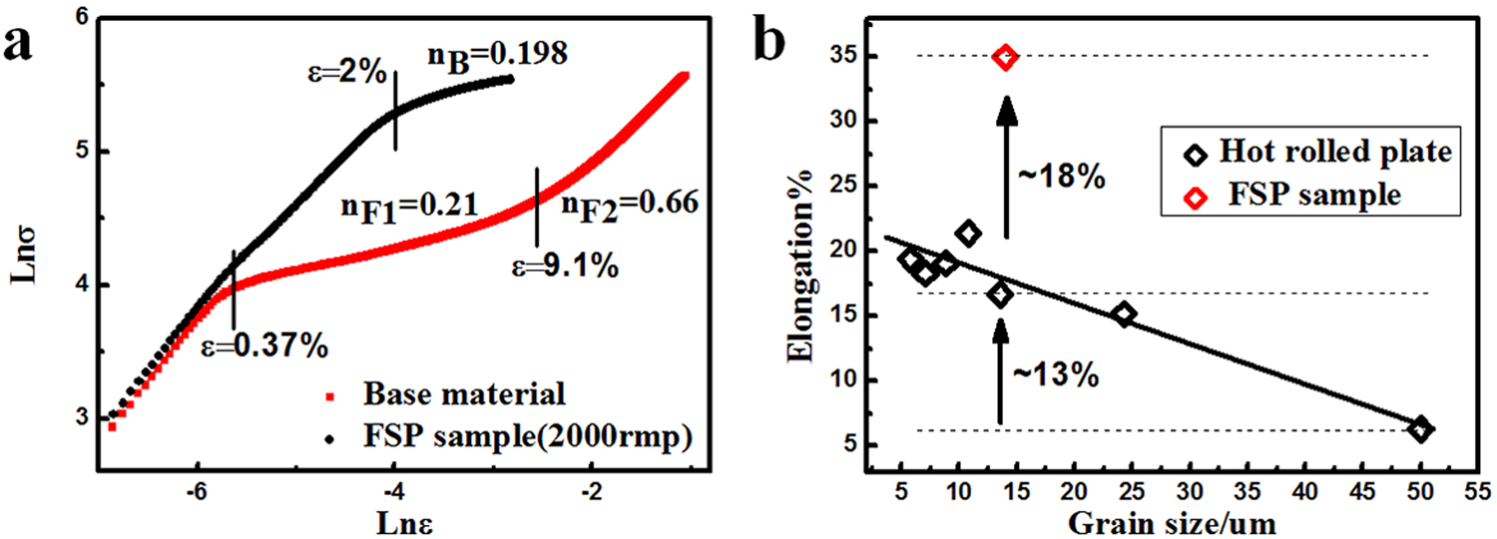
(**a**) *Lnσ* − *Lnε* curves (plotted depending on *σ* = *kε*^*n*^ where the slope represent the strain hardening exponent n) and (**b**) the variation of elongation against grain size of FSPed sample (2000 rpm) and hot-rolled plates.

To compare the difference, the strain hardening exponent- n of base material and FSPed sample (2000 rpm–60 mm/min) was calculated as the slope of the $\mathrm{ln}\,{\rm{\sigma }}-\,\mathrm{ln}\,{\rm{\varepsilon }}$ curves, as shown in Fig. 10a. The initial linear stage which corresponded to elastic strain range showed similar slopes for both the base and FSP material. However the FSPed sample shows two different strain hardening stages, while only one single strain- hardening stage was seen for the base material. Although the yield stress was somewhat lower for FSPed sample, the following strain hardening effect showed a similar level to base material. With further strain, there was a sudden increase in strain hardening exponent, where a rather high n value was found. This might imply different plastic mechanisms that operated at higher strain range. Since the basal poles were tilted to PD, which was also the loading direction during tensile tests, it was reasonable to think that basal slip was easily activated during initial strain range. On further straining, with the extensive propagation of dislocations and the potential redeveloped basal texture, other plastic mechanism such as contraction twinning and $\mathop{a}\limits^{\rightharpoonup }+\mathop{c}\limits^{\rightharpoonup }$ slip would be involved, which might lead to a significant increase of work hardening exponent.

### Comparative analyzes between FSPed and strong textured rolled plate

**Figure 11:**
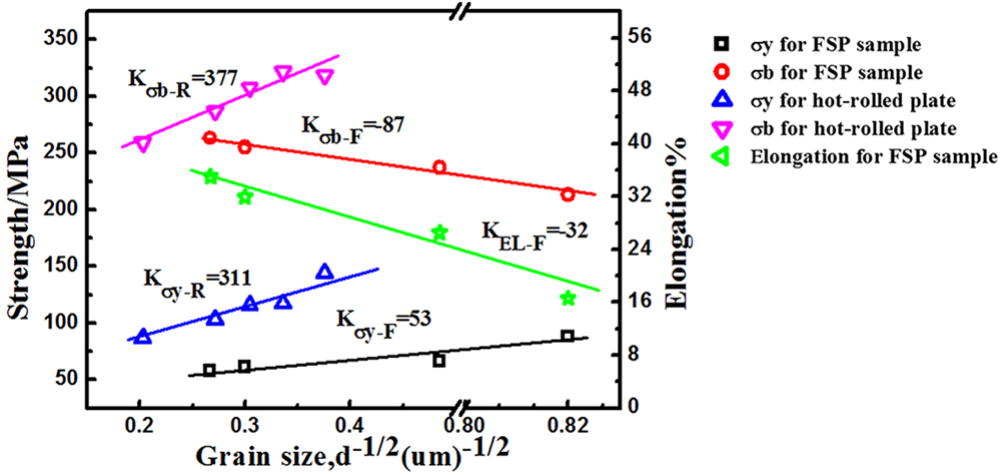
The variation in elongation and strength against grain size^−1/2^ for FSPed samples and hot-rolled plates.

Since it was found that two important factors, texture and grain size, were altered after FSP, the observed variation in strength, ductility as well as strain hardening behavior during this process was believed to originate from them. To separate the effect of these two factors, tensile tests were also conducted on strongly textured specimens (Fig. 7c) with different grain sizes for comparison. As reported, the texture underwent little change during annealing below 550 °C^32,33^. Therefore, the static annealing for the hot-rolled plate was conducted at 300–500 °C for 3 h, the grain size distribution was shown in Fig. 8b, increasing from 5.7 to 24.2 um. The true stress strain curves were shown in Fig. 9b. It could be seen that with strong basal texture, both the yield stress and elongation would increase with the decrease of grain size. This means that the grain refinement did improve both the strength and ductility for the experimental material. However it needed to be mentioned that the ductility improvement from grain refinement was not so effective in rolled sheets compared with FSP, when the basal texture was weakened by tilting towards PD direction. The expected increment in tensile elongation would be less than 15% for grain refinement effect^34–36^; the extra bonus of 18%, as indicated in Fig. 10b, should come from the other factor - texture. With a strong basal texture, both the yield stress and ultimate stress would increase with the refinement of grains, complying with Hall-Petch mechanism. However such relationship failed when the basal texture was altered by FSP, where the yield stress was about 1/2 of that of a rolled sheet with similar grain size as shown in Fig. 11. When the basal texture was weakened, by tilting towards PD, basal slip was more easily activated during tensile test which would be the main reason for the lower yield strength.

In condition of a strong texture, on the other hand, the rolled sheets showed a gradual increase in elongation with the refinement of grains. This grain size dependent ductility could be attributed to the competition between $\mathop{a}\limits^{\rightharpoonup }+\mathop{c}\limits^{\rightharpoonup }$ slip and contraction twinning, both of which accommodated compression along c-axis for basal textured polycrystalline of magnesium. It has been frequently reported, also by our previous work, that contraction twinning would be suppressed by grain size refinement; and instead $\mathop{a}\limits^{\rightharpoonup }+\mathop{c}\limits^{\rightharpoonup }$ slip would be more easily and homogeneously activated with the help of plastic compatibility stress from grain boundaries^37^. In this way, the ductility could be improved by grain refinement in condition of a basal texture. However, the enhancement in plasticity were much more significant when basal texture was weakened by FSP. The net effect was estimated to be 18%, by subtracting the influence of grain refinement. In general for metallic materials, grain refinement would improve the plasticity by enhancing the homogeneity of plastic deformation^38^. This was true for the experimental material with strong basal texture. For FSPed samples with inclined texture, however, although the elongation was usually higher than strongly basal textured condition, an evident loss in plasticity was clearly seen when grain size was reduced.

**Figure 12:**
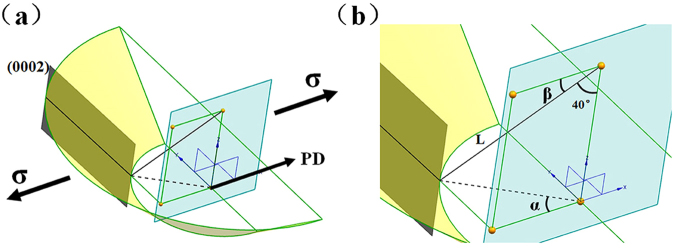
(**a**) Schematic drawing showing the (0002) slip planes distribution in the stir zone and (**b**) the magnified schematic showing the angle α and β (the line L was the normal of (0002) plane).

Concerning the strength, although the yield strength *σ*_*y*_ suffered a quick drop after FSP due to texture effect, for FSPed samples with modified texture, the yield strength also complied with Hall-Petch relationship, with a lower *K* value however (Fig. 11). As reported^28^, the reduced *K* value (*K* = 160 MPa·um^−1/2^) for FSPed samples (soft orientation) was contributed to the high Schmid factor ($m=\frac{2}{\pi }{\int }_{0}^{\pi /2}\frac{3}{\pi }\cos \,\chi d\chi \approx 0.3,$ the *χ* was the angle between loading direction and (0002) plane) of basal slip. In this study, the orientation Schmid factor of base material with strong basal texture was nearly 0. For the FSPed sample, in consideration of the conical pin of stirring tool and the weak basal texture (inclining 30°–50° from ND to PD-TD plane, in this study the average inclined angle was regarded as 40°), the (0002) basal planes could be roughly considered to be a conical surface surrounded the tool pin in the stir zone, as shown in Fig. 12. For any value of *α*, there exist a corresponding (0002) basal plane and the angle between the (0002) plane and loading direction was (90° − *β*). Therefore, the orientation Schmid factor *m* as a function of *β* could be written as^28^

1 $$m(\beta )=\frac{3}{\pi }\,\cos (90^\circ -\beta )\sin (90^\circ -\beta )=\frac{3}{\pi }\,\sin \,\beta \,\cos \,\beta $$

According to the geometrical relationship between *α* and *β*, the relationship between *α* and *β* could be expressed as

2a $$\sin \,\beta =\,\sin \,40^\circ \sqrt{{\sin }^{2}\alpha +{(1+\tan \alpha )}^{2}},$$

2b $$\cos \,\beta =\,\sin \,{\rm{40}}^\circ \,\cos \,\alpha .$$

So the orientation Schmid factor *m* as a function of *α* could be written as

3 $$m(\alpha )={\sin }^{2}40^\circ \,\cos \,\alpha \sqrt{{\sin }^{2}\alpha +{(1+\tan \alpha )}^{2}}.$$

Note that the angle of *α* varies from 0° to 90°, the mean orientation factor could be obtained as follows:

4 $$m=\frac{2}{\pi }{\int }_{0}^{2\pi }\frac{3}{\pi }{\sin }^{2}40^\circ \,\cos \,\alpha \sqrt{{\sin }^{2}\alpha +{(1+\tan \alpha )}^{2}}d\alpha \approx \mathrm{0.33.}$$

So the Schmid factor of basal slip in this study was 0.33 which was higher than that obtained by Y.N. Wang *et al*.^28^. This meant that the resistance of the grain boundaries as an obstacle to slip across the grain boundary was lower and resulted in lower grain size dependence (lower *K* value).

On the other hand, the ultimate tensile strength *σ*_*b*_ of FSPed samples abnormally got decreased with refining grain size and resulted in a negative *K* value (Fig. 11). This was consistent with the trend of elongation with grain size for FSPed samples, which implied a possible relationship between elongation and *σ*_*b*_. Indeed, with inclined basal texture, FSPed samples have a nearly constant strain hardening rate after yielding and the grain size dependence of yield strength was weak, so a higher *σ*_*b*_ would be always obtained for coarser grains with greater elongation.

## Conclusions

In summary, FSP caused significant grain refinement and modified the basal texture for the experimental AZ31 magnesium sheets. However the grain size did not show much variation within the processed region, the observed irregular hardness distribution in FSP region was attributed to local micro-texture effect based on EBSD results. Uniaxial tensile tests were conducted on both FSPed and rolled samples with varying grain sizes for comparative analysis. With strong basal texture, both the strength and ductility was improved by grain refinement. This grain size dependent ductility improvement could be attributed to the growing superiority of $\mathop{a}\limits^{\rightharpoonup }+\mathop{c}\limits^{\rightharpoonup }$ slip to contraction twinning with grain refinement. The ductility improvement was more evident when texture was modified by FSP, meanwhile the yield strength showed a lower Hall-Petch coefficient than in basal textured condition. The ultimate tensile strength *σ*_*b*_ abnormally got decreased with refining grain size, which was consistent with the trend of elongation.
